# MEAHNE: miRNA–Disease Association Prediction Based on Semantic Information in a Heterogeneous Network

**DOI:** 10.3390/life12101578

**Published:** 2022-10-11

**Authors:** Chen Huang, Keliang Cen, Yang Zhang, Bo Liu, Yadong Wang, Junyi Li

**Affiliations:** 1 School of Computer Science and Technology, Harbin Institute of Technology (Shenzhen), Shenzhen 518055, China; 20s151107@stu.hit.edu.cn (C.H.); 21s151104@stu.hit.edu.cn (K.C.); ydwang@hit.edu.cn (Y.W.); 2 College of Science, Harbin Institute of Technology (Shenzhen), Shenzhen 518055, China; zhangyang07@hit.edu.cn; 3 Center for Bioinformatics, School of Computer Science and Technology, Harbin Institute of Technology, Harbin 150001, China; bo.liu@hit.edu.cn; 4 Guangdong Provincial Key Laboratory of Novel Security Intelligence Technologies, Harbin Institute of Technology (Shenzhen), Shenzhen 518055, China

**Keywords:** heterogeneous network, miRNA–disease association, semantic information, attention aggregation

## Abstract

Correct prediction of potential miRNA–disease pairs can considerably accelerate the experimental process in biomedical research. However, many methods cannot effectively learn the complex information contained in multisource data, limiting the performance of the prediction model. A heterogeneous network prediction model (MEAHNE) is proposed to make full use of the complex information contained in multisource data. To fully mine the potential relationship between miRNA and disease, we collected multisource data and constructed a heterogeneous network. After constructing the network, we mined potential associations in the network through a designed heterogeneous network framework (MEAHNE). MEAHNE first learned the semantic information of the metapath instances, then used the attention mechanism to encode the semantic information as attention weights and aggregated nodes of the same type using the attention weights. The semantic information was also integrated into the node. MEAHNE optimized parameters through end-to-end training. MEAHNE was compared with other state-of-the-art heterogeneous graph neural network methods. The values of the area under the precision–recall curve and the receiver operating characteristic curve demonstrated the superiority of MEAHNE. In addition, MEAHNE predicted 20 miRNAs each for breast cancer and nasopharyngeal cancer and verified 18 miRNAs related to breast cancer and 14 miRNAs related to nasopharyngeal cancer by consulting related databases.

## 1. Introduction

miRNA is a type of noncoding RNA that plays an important role in the regulation of gene expression in eukaryotes [1,2,3]. The important roles of miRNAs in the occurrence and development of diseases have been revealed through the continuous improvement of biological technology [4,5,6]. During the development of diseases, miRNA can inhibit or promote disease by interacting with miRNA targets [7,8]. Identifying the miRNAs related to a disease is of great help for prevention and diagnosis. However, the number of elements in the existing miRNA set is much larger than the number of miRNAs associated with diseases, representing a considerable challenge in biomedical research. Therefore, computational methods are used to predict the links between miRNAs and diseases. The computational research methods used to predict the association between miRNA and disease can be divided into three categories: prediction based on similarity measures, machine-learning-based methods, and graph-neural-network-based methods.

The central idea of the method based on similarity measures is that miRNAs with similar functions may be associated with similar diseases. Jiang et al. [9] established an miRNA functional similarity matrix and an miRNA–disease adjacency matrix to form a network and calculated the similarity score in the network. Chen et al. [10] designed a prediction model that integrated miRNA functional similarity, disease semantic similarity, and Gaussian interaction profile kernel similarity between disease and miRNA. After the multisource matrices were fused, they calculated within-score and between-score differences between miRNA and diseases to make predictions. Chen et al. [11] regarded disease-related miRNAs as seeds and used these seeds as starting points to perform a restarting random walk on the miRNA functional similarity network. In order to alleviate the problem of sparse connections in the similarity network, You et al. [12] enriched the edges of the network by using matrix completion method. This method used a depth-first search algorithm to obtain potential miRNA–disease associations while walking the network.

Since their development, machine learning methods have been widely used in biomedical research [13,14,15]. Wu et al. [16] built and optimized an miRNA–disease adjacency matrix and used the collaborative matrix decomposition method to obtain a representation matrix of miRNA and disease. Chen et al. [17] combined miRNA functional similarity, disease semantic similarity, and Gaussian interaction profile kernel similarity calculations into the comprehensive similarity of miRNA and disease. They added the similarity into the miRNA–disease adjacency matrix and decomposed the adjacency matrix. Xu et al. [18] established an miRNA target regulatory network and input the miRNA features into a support vector machine (SVM) for prediction. Xuan et al. [19] used family information as an important factor for prediction and proposed that miRNAs in the same family may be associated with the same disease. Pasquier et al. [20] fused miRNA-related information and proposed a vector space model to predict miRNA–disease associations. Luo et al. [21] recently proposed a model called KRLSM, which fuses multiple omics data sources and used Kronecker RLS to make predictions.

In graph-neural-network-based methods, miRNAs and diseases are built into a graph network, and a graph neural network (GNN) is used to extract structural information in the network [22,23,24,25]. GCN [22] obtains the representation of nodes in space by aggregating neighbor nodes in the spatial domain and using nonlinear activation functions. GAT [23] proposes that different neighbor nodes in the spatial domain have different importance to the target node, whereas the importance of different nodes is obtained by using the attention mechanism. Simonovsky et al. [26] used a multilayer neural network to represent nodes as low-dimensional vectors and a decoder to decode the low-dimensional vectors into node representations. Li et al. [27] established an miRNA functional similarity matrix and disease semantic similarity matrix into a graph and used GCN to learn the structural information of the graph. Structural information was fed into a multilayer neural network to obtain representations of nodes. To effectively integrate heterogenous miRNA and disease information, Li et al. [28] designed a graph encoder that contains an aggregator function and a multilayer perceptron that aggregates node neighborhood information to generate a low-dimensional embedding of miRNAs and diseases.

Homogeneous graph neural networks ignore the semantic information contained between different types of nodes. A heterogeneous graph neural network is able to learn semantic information in the network very well. Metapath2vec [29] introduced the concept of metapath into graph representation learning. Metapath2vec samples multiple sequences composed of nodes from heterogeneous networks through the metapath setting. This word representation learning model processes sequences into low-dimensional vector representations. Wang et al. [30] processed a heterogeneous graph into multiple subgraphs and used an attention mechanism to learn representation of nodes from each metapath. They also used semantic-level attention to integrate the representations from multiple metapaths. However, when selecting subgraphs according to the metapath, such models ignore the intermediate nodes on the metapath, resulting in the loss of information. This problem is also called the early-summarization problem [31]. Fu et al. [32] fused the node vector on the metapath instances into the target node by spatial rotation. In this way, nodes can learn rich semantic information. However, indiscriminately aggregating different types of nodes can make node embeddings too similar.

Here, we propose a new semantic-based attention mechanism for use on heterogeneous graphs; we applied the proposed mechanism to predict potential miRNA–disease connections in heterogeneous networks. We first collected multisource data to form a heterogeneous network. We used metapaths to split the original graph into multiple subgraphs. Then, a nonlinear neural network was used to mine the semantic information contained in the metapath instances in the subgraphs, which learned the diverse semantic information from different metapath modes. The obtained semantic information was encoded into association weights through the attention mechanism. The target node aggregates the information of its metapath neighbors through association weights. Finally, the representations of target nodes under multiple metapaths are fused through a nonlinear neural network. This model can make good use of metapaths to learn the complex association information of multisource biological networks.

## 2. Materials and Method

### 2.1. Data Collection and Construction of Heterogeneous Networks

In this section, we introduce the data we used, which consist of three types of nodes, namely miRNA, disease, and gene nodes, and four types kinds of relationships between the three types of nodes. The four types of relationships are miRNA–disease relationships, miRNA–gene relationships, disease–gene relationships, and protein–protein interaction relationships (Table 1 and Table 2).

We collected related links between miRNAs and diseases from the HMDD3.2 [33] database. HMDD is a reliable database that specifically collects miRNA–disease associations. We collected 17,972 links between 1206 miRNAs and 893 diseases and integrated miRNAs and diseases as nodes and miRNA–disease associations as edges into the heterogeneous network. We collected related links between miRNAs and target genes from the Circ2disease [34] database. We selected 4676 links between 202 miRNAs and 1713 genes and integrated miRNAs and target genes as nodes and the associations between them as edges into the heterogeneous network. We collected the related links between diseases and genes from DisGeNET [35]. We selected 84,038 links between 11,181 diseases and 9703 genes and integrated diseases and genes as nodes and the associations between them as edges into the heterogenous network.

When constructing the PPI network, we used the PPI network data retrieved directly from HerGePred [36]. We selected the genes that are related to miRNAs and disease. The 105,171 associations between these genes were integrated into the heterogeneous network as edges. Finally, we established a heterogeneous network with 1296 miRNAs, 11,783 diseases, 10,116 genes, and 211,857 edges.

### 2.2. Methods

#### 2.2.1. Related Definitions

Heterogeneous networks have many types of nodes and many types of relationships. The paths composed of different types of nodes and different types of instances contain rich semantic information, which is not available in homogeneous graphs. To learn the semantic information in heterogeneous graphs, the concept of a metapath is proposed. For example, ${𝓅}_{1}={\;a}_{1}\frac{r_{1}}{\to }a_{2}\frac{r_{3}}{\to }a_{3}\frac{r_{5}}{\to }a_{1}$ is a kind of metapath, and ${𝓅}_{2}={\;a}_{2}\frac{r_{3}}{\to }a_{3}\frac{r_{4}}{\to }a_{2}$ is another kind of metapath. $\mathrm{In}\;{𝓅}_{i}({𝓅}_{i}\in P)$ , ${𝓅}_{i}$ represents a specific metapath, and P represents all types of metapaths in the heterogeneous graph. For $a_{i}\in A\;\mathrm{and}\;r_{i}\in ℛ$ , A represents the collection of all node types in the heterogeneous graph, and ℛ represents the collection of all relationship types in the heterogeneous graph.

In this experiment, we used multiple metapaths to mine heterogeneous networks. The original network was sampled under each metapath to obtain subgraphs. We called all the node sequences on the subgraph that conformed to the metapath mode metapath instances. For example, $v_{a_{1}}^{1}\to v_{a_{2}}^{5}\to v_{a_{3}}^{3}\to v_{a_{5}}^{2}$ is a metapath instance under ${𝓅}_{1}$ in which $v_{a_{i}}^{i}$ represents the ith node of type $a_{i}$ .

The sampling subgraph under each metapath contained the target node and the metapath instance connected to the target node. We called the nodes on the subgraph that are of the same type as the target node metapath neighbors.

#### 2.2.2. Specific Steps

**Figure 1:**
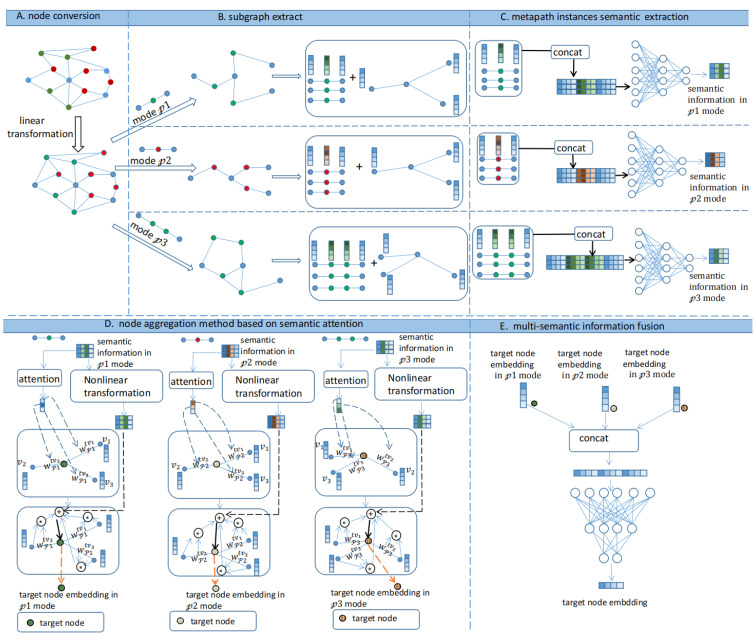
MEAHNE framework. A. Nodes of different types are projected into the same space. B. The subgraph under each metapath and the metapath edges on subgraphs are extracted. C. We encoded the semantic information into values as semantic weights to aggregate nodes of a single type. D. The semantic information on metapath edges was aggregated to obtain a more powerful node representation. E. Representations under all metapaths were fused to obtain the final node embedding.

In this section, we introduce the main methods, ideas, and specific implementation details of the MEAHNE model. The MEAHNE model is divided into six parts: A. node conversion, B. subgraph extraction, C. metapath instances semantic extraction, D. node aggregation method based on semantic attention, E. multisemantic information fusion, and F. link prediction. Figure 1 shows the overall framework of MEAHNE.

A. Node conversion

If we want to learn representations of heterogeneous networks, we need to perform interactive calculations on the nodes of the graph. However, heterogeneous graphs have multiple types of nodes, and different types of nodes are located in different spaces. If the nodes are not processed, interactive calculation between nodes becomes too difficult, so we first converted all types of nodes into the same space to facilitate calculations between nodes as follows.

A trainable linear transformation matrix was set for each type of node, and original nodes of different types were projected into the same space, as shown in Formula (1):

(1) $$h_{a_{i}}=M_{a_{i}}\cdot x_{a_{i}}$$

where $x_{a_{i}}$ represents the original feature vector of node type $a_{i}$ ; and $M_{a_{i}}\in {\mathbb{R}}^{d^{\prime }\times d_{a_{i}}}$ , in which $d^{\prime }$ represents the feature space dimension after space conversion, and $d_{a_{i}}$ represents the original feature dimension of node type $a_{i}$ .

B. Subgraph extraction

To mine heterogeneous graphs in multiple metapaths, the first step is to separate the corresponding subgraphs based on specific metapaths.

We separated the subgraph ( $G_{{𝓅}_{i}}$ ) according to the metapath ( ${𝓅}_{i}$ ); $G_{{𝓅}_{i}}$ represents the subgraph mined in ${𝓅}_{i}$ mode. The node sequence corresponding to ${𝓅}_{i}$ mode in $G_{{𝓅}_{i}}$ was sampled and denoted as P(v,u), which connects the target node (*v*) and its metapath neighbor (*u*).

C. Metapath instances semantic extraction

When mining the information from the corresponding subgraph ( $G_{{𝓅}_{i}}$ ) under a single metapath ( ${𝓅}_{i}$ ), different types of nodes are transformed into the same space through space conversion, which allows different types of nodes to represent each other. The metapath instance is composed of different types of nodes connected to each other and contains rich semantic information. Therefore, to learn the semantic information on the metapath instance in the subgraph, we first integrated the information on the metapath instance. Each metapath instance was represented as a vector that represents the semantic information on the instance. All the nodes on the metapath instance were concatenated according to the order of the metapath, as shown in Formula (2):

(2) $$h_{P\left(v,u\right)}=∥\left(P\left(v,u\right)\right)={∥}_{\forall t\in \left\{m^{P\left(v,u\right)}\right\}}\left(h_{t}\right)$$

where P(v,u) represents the metapath instance from v to u , $m^{P\left(v,u\right)}$ represents the set of nodes on the metapath instance, and $\;h_{P\left(v,u\right)}$ represents the vector obtained by concatenating the vectors of the nodes on the metapath instance ( P(v,u) ).

A nonlinear neural network was used to learn vector *h*, resulting in semantic information of the metapath instance. A nonlinear neural network, which has strong information extraction capabilities, is a network composed of multiple fully connected layers and nonlinear activation functions, as shown in Formula (3):

(3) $$\phi_{{𝓅}_{i}}^{l}=relu\left(W_{{𝓅}_{i}}^{\left(l\right)}relu\left(\cdots relu\left(W_{{𝓅}_{i}}^{\left(1\right)}X+b_{{𝓅}_{i}}^{1}\right)\cdots \right)+b_{{𝓅}_{i}}^{l}\right)$$

where $W_{{𝓅}_{i}}^{j}$ represents the weight matrix of the *j*th fully connected layer of the neural network under metapath ${𝓅}_{i}$ , the bias value of the *k*th layer of the neural network under metapath ${𝓅}_{i}$ is $b_{{𝓅}_{i}}^{k},X$ represents the input feature, and $\phi_{{𝓅}_{i}}^{l}$ represents the vector representation of input vector X learned through l connection layers in the neural network under metapath ${𝓅}_{i}$ . We used vector $h_{P\left(v,u\right)}$ as the input of the nonlinear neural network to obtain the semantic information of the metapath instance, as shown in Formula (4):

(4) $$h_{P\left(v,u\right)}^{\prime }=\phi_{𝓅}^{l}\left(h_{P\left(v,u\right)}\right)$$

D. Node aggregation method based on semantic attention

After obtaining the semantic information from the metapath instances, we can aggregate the semantic information into the target nodes connected to these metapath instances; the semantic information is obtained by the fusion of different types of nodes. If the target node only aggregates semantic information, each type of node contains information about other types of nodes, causing different types of nodes to lose their distinction. To make the node representation more complete based on the aggregation of semantic information, we aggregated the same types of nodes, and the embeddings obtained for different types of nodes were strongly distinguishable. For aggregating nodes of the same type, we designed a method to encode semantic information into attention weights and used the obtained attention coefficient to aggregate metapath neighbors. Finally, we fused the information obtained by the aggregation of nodes of the same type and semantic information from metapath instances as the final node representation.

**Figure 2:**
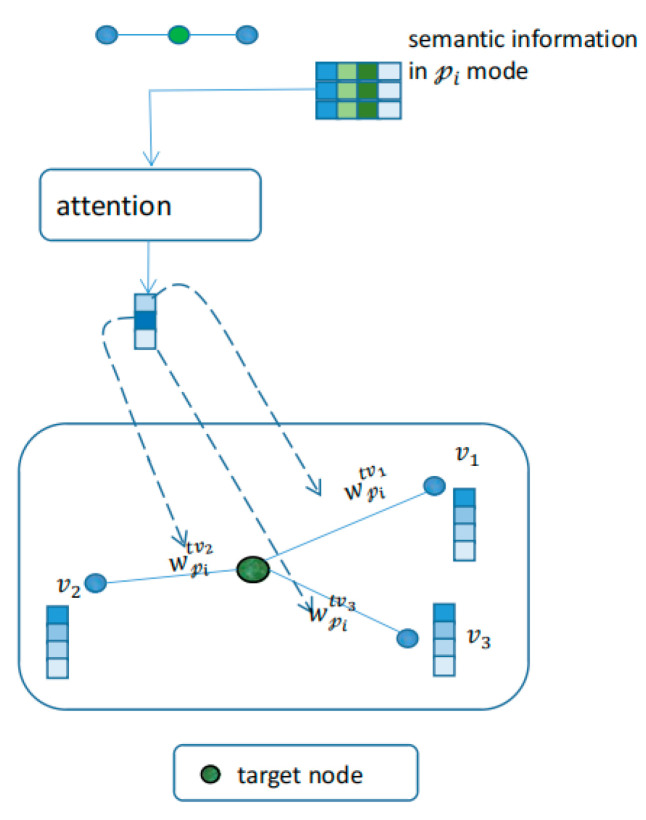
Encoding semantic information on the metapath instances into attention weights.

The metapath subgraph retains only the nodes of the same type as the target node to form a homogenous graph ( G ). Therefore, graph G only contains the target node and metapath neighbor of the target node. We encoded the semantic information on the instance using the attention mechanism as a weight value—the correlation strength coefficient between the target node and the metapath neighbor, as shown in Figure 2 and Equations (5) and (6).

(5) $$e_{𝓅}^{vu}=Leaky\_relu\left(a^{𝓅}\cdot h_{P\left(v,u\right)}^{\prime }\right)$$

(6) $$w_{𝓅}^{vu}=soft\_max\left(e_{𝓅}^{vu}\right)=\frac{\;exp\left(e_{𝓅}^{vu}\right)}{\sum_{s\in N_{v}^{𝓅}\;}exp\left(e_{𝓅}^{vs}\right)}$$

$e_{𝓅}^{vu}$ where represents the value encoded by the attention mechanism;  Leaky_relu () is a nonlinear activation function; $\;a^{𝓅}$ represents the attention weight matrix under metapath 𝓅 ; $N_{v}^{𝓅}$ represents the set of metapath neighbors connected to the target node (*v*) on the subgraph in mode 𝓅 ; and $w_{𝓅}^{vu}$ represents the semantic weight between node *v* and node u , where node u is the metapath neighbor of node v .

Next, the metapath neighbors of the same type were aggregated according to the weight ( $w_{𝓅}^{vu}$ ). The semantic information was also integrated to ensure the integrity of the node embedding.

To reasonably integrate semantic information during the node aggregation stage, we performed secondary learning on semantic information by continuously adjusting the proportion of semantic information through end-to-end optimization and by adaptively learning the optimal semantic information. We designed a trainable matrix to optimize the weights of semantic information and added nonlinear activation operations to the optimization results, as shown in Formula (7).

(7) $$h_{P\left(v,u\right)}^{\prime \prime }=relu\left(b^{𝓅}\cdot h_{P\left(v,u\right)}^{\prime }\right)$$

where $b^{𝓅}$ represents a learnable weight matrix under metapath 𝓅 , and the content of semantic information is continuously adjusted through end-to-end learning.

We used the learned metapath semantic weight to aggregate the metapath neighbors and added the semantic information learned twice. Therefore, the target node could be more comprehensively expressed, as shown in Formula (8):

(8) $$h_{v}^{𝓅}=h_{P\left(v,u\right)}^{\prime \prime }+\sum_{u\in N_{v}^{𝓅}}\left(w_{𝓅}^{vu}\cdot h_{u}\right)$$

In this way, the target node not only learned the semantic information on the metapath instance but also learned the information obtained by the aggregation of nodes of the same type. The nodes of different types remained distinct, making the representation of the nodes more complete.

E. Multisemantic information fusion

In the above steps, we only learned the graph under a single metapath. Our model learned the graph in multiple metapath modes and generated the representation of the target node in multiple metapath modes. We used neural network methods to integrate node representations under multiple metapaths, as shown by Formula (9):

(9) $$h_{v}={∥}_{\forall {𝓅}_{i}\in 𝓅}\left(h_{t}^{{𝓅}_{i}}\right)$$

where $h_{v}^{{𝓅}_{i}}$ represents the embedding obtained by aggregating the target node (*v*) under metapath ${𝓅}_{i}$ , and $\;h_{v}\;$ represents the result of concatenating the representation of the target node (*v*) under all metapaths. Then, the embedding ( $h_{v}$ ) was input into the nonlinear neural network to learn a low-dimensional embedding that fuses the target node representation under multiple metapaths, as shown in Formula (10):

(10) $$H_{v}\;=\;\phi \left(h_{v}\right)\;$$

After learning through a nonlinear neural network, $H_{v}$ represented a low-dimensional embedding that fused multiple metapath representation results as the final representation of the target node.

F. Link prediction

The vector inner product was used as the score of the link strength of the two nodes. If the two vectors are highly correlated, then the score of the node inner product will be higher. We used this as the basis for link prediction, as shown in Formula (11):

(11) $${\mathrm{score}}_{\mathrm{md}}=\sigma \left(<{ℋ}_{m},{ℋ}_{d}>\right)$$

Our link prediction was between miRNA and disease. The higher the prediction score, the stronger the correlation, and the lower the prediction score, the weaker the correlation. We used two-class cross entropy as the optimization target. Our optimization goal is shown in Formula (12):

(12) $$Loss=-\sum_{\left(m,d\right)\in \Phi }log\left(\sigma \left(<{ℋ}_{m},{ℋ}_{d}>\right)\right)-\sum_{\left(m,d\right)\in \Phi^{-}}log\left(\sigma \left(-<{ℋ}_{m},{ℋ}_{d}>\right)\right)$$

where Φ represents the set of miRNA and disease pairs that have been verified to be associated, and $\Phi^{-}$ represents the set of all miRNA–disease pairs that have not been experimentally verified. The goal of optimization is to increase the score between verified node pairs and decrease that between unverified node pairs. Because our model is an end-to-end training model, the parameters in the model are continuously optimized during the training process, and the continuously optimized parameters enable us to achieve the optimization goal.

## 3. Results and Discussion

### 3.1. Experimental Data and Performance Evaluation

We built miRNAs, diseases, and genes into a network and conducted experiments to compare our model with other comparative models on the network. The links that are verified from the databases in our dataset are positive samples, and the others are negative samples. We split the dataset into training (70%), validation (10%), and test (20%) sets using a random sampling method without repetition. The ratio of positive-to-negative samples in all sets is 1:1. Parameters of our model were set as follows: learning rate, 0.005; dropout rate, 0.5; network node dimension, 90; number of layers for semantic extraction, 1; number of neighbor samples, 60. To prevent the model from overfitting, we used an early stopping mechanism and set the patience of the mechanism to 3. We compared our method with other heterogeneous network embedding methods under three metrics: area under the receiver operating characteristic curve (AUC), area under the precision–recall curve (AP), and the prediction accuracy of the highest K in the prediction results (Precision@K). 

### 3.2. Factors Influencing Model Performance

**Figure 3:**
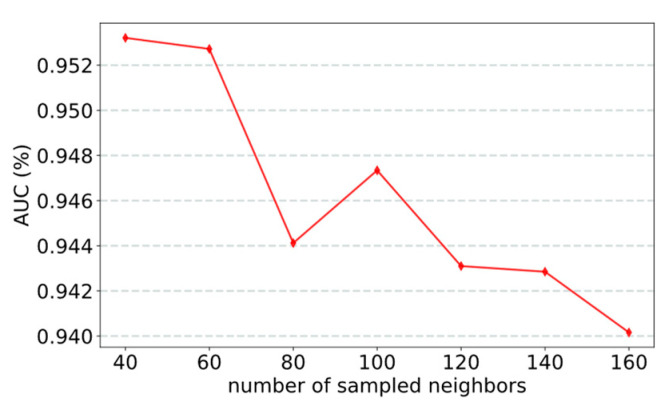
AUC obtained by the model sampling different numbers of neighbors.

**Figure 4:**
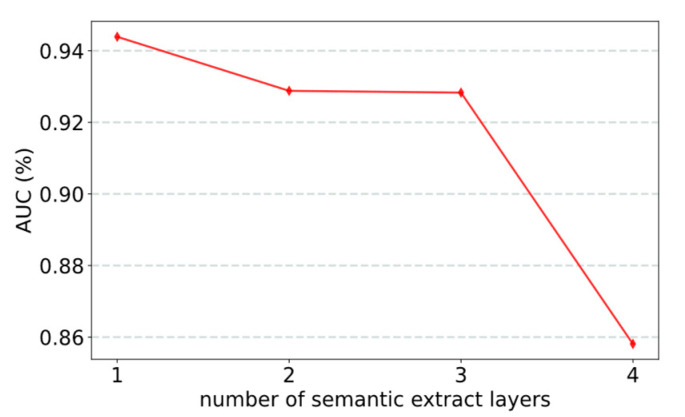
AUC obtained by the model with different numbers of semantic extraction layers.

Two factors significantly affect model performance: the number of sampled neighbors and the number of semantic extraction layers. The experimental results show that when the number of sampled neighbors is 40 (Figure 3) and there is one semantic extraction layer (Figure 4), the model achieved the best performance. In the experiment, we used the control variable method to evaluate the effect of parameters on the model by changing one parameter and keeping the other parameters fixed.

#### 3.2.1. Effect of the Number of Sampled Neighbors

Some nodes in the network have many neighbors, whereas others have few neighbors. If a node aggregates all its neighbors, some nodes receive too much information and other nodes receive too little information. This can considerably affect the predictive performance of the model. To solve this problem, our model adopts a random sampling method. Each node samples a fixed number of neighbors. In this way, the information of all nodes is relatively balanced, which can considerably improve the effect of the model. We analyze the effect of sampling number on the model by modifying the number of node-sampling neighbors (Figure 3). Experimental results show that our model performed best when the number of neighbors is 40 because sampling 40 neighbors can ensure that each node has enough neighbors to be sampled. If too few neighbors are sampled, the performance of the model will suffer from a lack of information.

#### 3.2.2. Effect of Number of Semantic Extract Layers

Assigning semantic attention weights to nodes is a key feature of the model. Semantic information directly affects the size of semantic attention weights. The number of layers of semantic information extraction affects the performance of the model. If the number of extraction layers is large, an overfitting effect is easily produced, resulting in partial loss of semantic information. The experimental results confirmed this (Figure 4). The model performs best with one extraction layer.

#### 3.2.3. Comparison with Other Models

**Figure 5:**
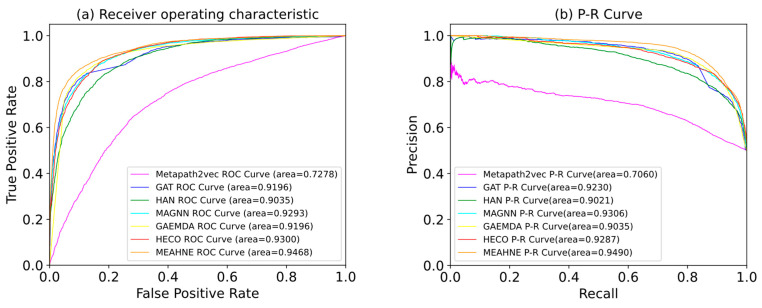
ROC and PR curves for all models: (**a**) ROC curves of all models; (**b**) P-R curves of all models.

**Figure 6:**
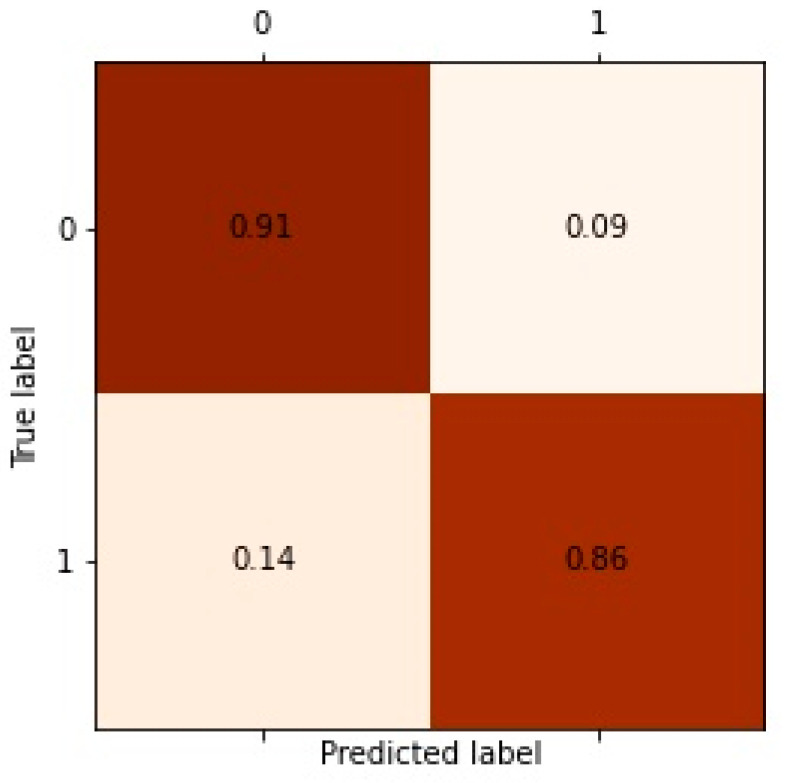
Confusion matrix of MEAHNE prediction result.

Comparison experiments have been conducted using the representative graph representation method in recent years; the heterogeneous graph representation method metapath2vec and the best performing matepath (miRNA–disease–gene–miRNA) are selected after multiple experiments. Because GAT is a homogeneous network method, we used metapaths to split the original network into homogeneous networks, used the GAT method to extract the information of homogeneous networks, and selected the best result as the performance of GAT model. HAN, MAGNN, HECO, and GAEMDA are all well-performing heterogeneous graph neural network methods. For the sake of fairness, we adjusted these models to the best results as the model effects. Our model achieved the best performance under both AUC and AP metrics (Table 3). The receiver operating characteristic (ROC) and precision–recall (P-R) curves are shown in Figure 5. The confusion matrix is shown in Figure 6.The codes of Metapath2vec, GAT, and HAN were derived from the open-source graph representation learning framework OpenHINE. The rest of the comparative test codes were retrieved from their official GitHub codes (Supplementary Materials).

### 3.3. Case Study

In order to verify the effectiveness of our model, we selected two cancers in the dataset to predict potential cancer-associated miRNAs. The model predicted 18 validated breast-cancer-related miRNAs that were included in our dataset. The model predicted 14 validated nasopharyngeal-carcinoma-related miRNAs that were not included in our dataset, as shown in Table 4 and Table 5. “*” indicates that the miRNA predicted by the model has been verified in the dbDEMC database [38].

### 3.4. Ablation Experiment

**Figure 7:**
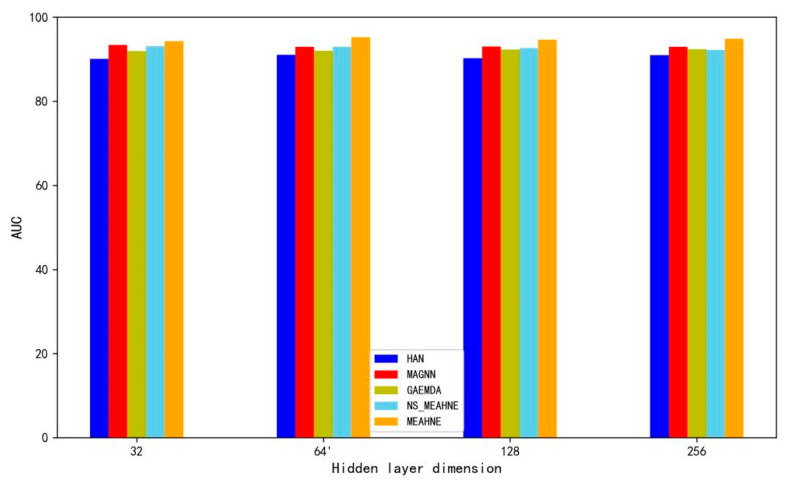
Result of ablation experiment.

In order to demonstrate the effectiveness of the semantic attention mechanism of our model, we removed the semantic attention module and replaced it with summation. Accordingly, we designed a comparative experiment, changing the hidden layer dimensions of the models and observing how the models performed. The experimental results are shown in Figure 7. The performance of our model diminished significantly without the use of a semantic attention module. The experimental results illustrate the effectiveness of the semantic attention module. NS_MEAHNE means MEAHNE without semantic attention module.

## 4. Conclusions

In this paper, we propose a heterogeneous graph neural network model that can fully learn a variety of information in a heterogeneous network. This model integrates the semantic information and node type information into the node representation, which not only avoids the early-summarization [25] problem but also avoids the problem of homogenization of different types of nodes due to a large amount of aggregated semantic information and maintains the distinction of nodes. We propose an attention mechanism based on the semantics of the metapath instance. Under each metapath, the semantic information of the learned metapath instance is encoded into attention weights to perform node aggregation, and the semantic information is also integrated into the node representation so that nodes retain comprehensive information. Finally, a multilayer neural network is used to fuse the representation of multiple metapaths as the final node representation. Experimental results show that our model performs better than other models.

However, there is still room for improvement with respect to our model. The semantic information obtained through the semantic information extraction layer considerably affects the allocation of attention weights, and we used a nonlinear neural network as the extraction tool. Whether other graph neural network methods can be used for semantic information extraction deserves further investigation.

## Figures and Tables

**Table 1:** Nodes in the network.

Node	Number	Source Dataset
miRNA	1296	HMDD3.2/Circ2disease
Disease	11,783	DisGeNET/HMDD3.2
Gene	10,116	Circ2disease/DisGeNET

**Table 2:** Relationships in the network.

Relationship	Number	Source
miRNA–disease	17,972	HMDD3.2 [33]
miRNA–gene	4676	Circ2disease [34]
Disease–gene	84,038	DisGeNET [35]
Gene–gene	105,171	HerGePred [36]

**Table 3:** Model evaluation.

Model	AUC	AP	P@500	P@1000	P@1500
Metapath2vec [29]	72.78	70.60	99.60	95.44	80.12
GAT [23]	91.96	92.30	96.53	94.25	90.31
HAN [30]	92.35	92.21	99.56	99.13	96.09
GAEMDA [28]	91.96	90.35	99.50	98.21	94.89
MAGNN [32]	92.93	93.06	99.32	98.10	94.28
HECO [37]	93.00	92.87	99.14	98.35	93.46
MEAHNE	95.20	95.82	99.65	98.85	96.45

**Table 4:** Breast cancer.

miRNA	Breast Cancer	miRNA	Breast Cancer
hsa-mir-143	*	hsa-mir-181b-2	*
hsa-mir-296	*	hsa-mir-29b-1	*
hsa-mir-192	*	hsa-mir-1-1	
hsa-mir-133a-1		hsa-mir-196a	*
hsa-mir-382	*	hsa-mir-148b	*
hsa-mir-34c	*	hsa-mir-26a-2	*
hsa-mir-224	*	hsa-mir-18	*
hsa-mir-497	*	hsa-mir-144	*
hsa-mir-149	*	hsa-mir-30d	*
hsa-mir-383	*	hsa-mir-218-1	*

**Table 5:** Nasopharyngeal carcinoma (NPC).

miRNA	NPC	miRNA	NPC
hsa-mir-126	*	hsa-mir-182	
hsa-mir-210		hsa-mir-196a	
hsa-mir-17	*	hsa-mir-34	
hsa-mir-503	*	hsa-mir-99a	*
hsa-mir-20a	*	hsa-mir-29b-1	*
hsa-mir-18a	*	hsa-mir-192	
hsa-mir-424	*	hsa-mir-215	
hsa-mir-221	*	hsa-mir-335	*
hsa-mir-375	*	hsa-mir-342	*
hsa-mir-150	*	hsa-mir-100	*

## Supplementary Materials

All additional files are available at: https://github.com/yyx-hc/MEAHNE.

## References

[1] Lee R.C., Ambros V. (2001). An Extensive Class of Small RNAs in Caenorhabditis Elegans. Science.

[2] Ambros V. (2004). The Functions of Animal MicroRNAs. Nature.

[3] Lee R.C., Feinbaum R.L., Ambros V. (1993). The C. Elegans Heterochronic Gene Lin-4 Encodes Small RNAs with Antisense Complementarity to Lin-14. Cell.

[4] Guo C., Sah J.F., Beard L., Willson J.K.V., Markowitz S.D., Guda K. (2008). The Noncoding RNA, miR-126, Suppresses the Growth of Neoplastic Cells by Targeting Phosphatidylinositol 3-Kinase Signaling and Is Frequently Lost in Colon Cancers. Genes. Chromosomes Cancer.

[5] Calin G.A., Croce C.M. (2006). MicroRNA Signatures in Human Cancers. Nat. Rev. Cancer.

[6] Cahill S., Smyth P., Denning K., Flavin R., Li J., Potratz A., Guenther S.M., Henfrey R., O’Leary J.J., Sheils O. (2007). Effect of BRAFV600E Mutation on Transcription and Post-Transcriptional Regulation in a Papillary Thyroid Carcinoma Model. Mol. Cancer.

[7] He L., Hannon G.J. (2004). MicroRNAs: Small RNAs with a Big Role in Gene Regulation. Nat. Rev. Genet..

[8] Goh J.N., Loo S.Y., Datta A., Siveen K.S., Yap W.N., Cai W., Shin E.M., Wang C., Kim J.E., Chan M. (2016). MicroRNAs in Breast Cancer: Regulatory Roles Governing the Hallmarks of Cancer. Biol. Rev. Camb. Philos. Soc..

[9] Jiang Y., Liu B., Yu L., Yan C., Bian H. (2018). Predict miRNA-Disease Association with Collaborative Filtering. Neuroinformatics.

[10] Chen X., Yan C.C., Zhang X., You Z.-H., Deng L., Liu Y., Zhang Y., Dai Q. (2016). WBSMDA: Within and between Score for miRNA-Disease Association Prediction. Sci. Rep..

[11] Chen X., Liu M.-X., Yan G.-Y. (2012). RWRMDA: Predicting Novel Human MicroRNA-Disease Associations. Mol. Biosyst..

[12] You Z.-H., Huang Z.-A., Zhu Z., Yan G.-Y., Li Z.-W., Wen Z., Chen X. (2017). PBMDA: A Novel and Effective Path-Based Computational Model for miRNA-Disease Association Prediction. PLoS Comput. Biol..

[13] Ahmadi M., Sharifi A., Jafarian Fard M., Soleimani N. (2021). Detection of Brain Lesion Location in MRI Images Using Convolutional Neural Network and Robust PCA. Int. J. Neurosci..

[14] Davoudi A., Ahmadi M., Sharifi A., Hassantabar R., Najafi N., Tayebi A., Kasgari H.A., Ahmadi F., Rabiee M. (2021). Studying the Effect of Taking Statins before Infection in the Severity Reduction of COVID-19 with Machine Learning. BioMed Res. Int..

[15] Experimental and Numerical Diagnosis of Fatigue Foot Using Convolutional Neural Network. https://pubmed.ncbi.nlm.nih.gov/34121524/.

[16] Wu T.-R., Yin M.-M., Jiao C.-N., Gao Y.-L., Kong X.-Z., Liu J.-X. (2020). MCCMF: Collaborative Matrix Factorization Based on Matrix Completion for Predicting miRNA-Disease Associations. BMC Bioinform..

[17] Chen X., Wang L., Qu J., Guan N.-N., Li J.-Q. (2018). Predicting miRNA-Disease Association Based on Inductive Matrix Completion. Bioinformatics.

[18] Xu J., Li C.-X., Lv J.-Y., Li Y.-S., Xiao Y., Shao T.-T., Huo X., Li X., Zou Y., Han Q.-L. (2011). Prioritizing Candidate Disease miRNAs by Topological Features in the miRNA Target-Dysregulated Network: Case Study of Prostate Cancer. Mol. Cancer Ther..

[19] Xuan P., Han K., Guo M., Guo Y., Li J., Ding J., Liu Y., Dai Q., Li J., Teng Z. (2013). Prediction of MicroRNAs Associated with Human Diseases Based on Weighted k Most Similar Neighbors. PLoS ONE.

[20] Pasquier C., Gardès J. (2016). Prediction of miRNA-Disease Associations with a Vector Space Model. Sci. Rep..

[21] Luo J., Xiao Q., Liang C., Ding P. (2017). Predicting MicroRNA-Disease Associations Using Kronecker Regularized Least Squares Based on Heterogeneous Omics Data. IEEE Access.

[22] Kipf T.N., Welling M. (2017). Semi-Supervised Classification with Graph Convolutional Networks. arXiv.

[23] Velikovi P., Cucurull G., Casanova A., Romero A., Liò P., Bengio Y. (2017). Graph Attention Networks. arXiv.

[24] Zhang J., Shi X., Xie J., Ma H., King I., Yeung D.-Y. (2018). GaAN: Gated Attention Networks for Learning on Large and Spatiotemporal Graphs. arXiv.

[25] Hamilton W.L., Ying R., Leskovec J. (2018). Inductive Representation Learning on Large Graphs. arXiv.

[26] Simonovsky M., Komodakis N. (2018). GraphVAE: Towards Generation of Small Graphs Using Variational Autoencoders. arXiv.

[27] Li J., Zhang S., Liu T., Ning C., Zhang Z., Zhou W. (2020). Neural Inductive Matrix Completion with Graph Convolutional Networks for miRNA-Disease Association Prediction. Bioinformatics.

[28] Li Z., Li J., Nie R., You Z.-H., Bao W. (2021). A Graph Auto-Encoder Model for miRNA-Disease Associations Prediction. Brief. Bioinform..

[29] Dong Y., Chawla N.V., Swami A. Metapath2vec: Scalable Representation Learning for Heterogeneous Networks. Proceedings of the 23rd ACM SIGKDD International Conference on Knowledge Discovery and Data Mining.

[30] Wang X., Ji H., Shi C., Wang B., Cui P., Yu P., Ye Y. (2021). Heterogeneous Graph Attention Network. arXiv.

[31] Qu Y., Bai T., Zhang W., Nie J., Tang J. (2019). An End-to-End Neighborhood-Based Interaction Model for Knowledge-Enhanced Recommendation. arXiv.

[32] Fu X., Zhang J., Meng Z., King I. MAGNN: Metapath Aggregated Graph Neural Network for Heterogeneous Graph Embedding. Proceedings of the Web Conference 2020.

[33] Huang Z., Shi J., Gao Y., Cui C., Zhang S., Li J., Zhou Y., Cui Q. (2019). HMDD v3.0: A Database for Experimentally Supported Human MicroRNA-Disease Associations. Nucleic Acids Res..

[34] Yao D., Zhang L., Zheng M., Sun X., Lu Y., Liu P. (2018). Circ2Disease: A Manually Curated Database of Experimentally Validated CircRNAs in Human Disease. Sci. Rep..

[35] Piñero J., Bravo À., Queralt-Rosinach N., Gutiérrez-Sacristán A., Deu-Pons J., Centeno E., García-García J., Sanz F., Furlong L.I. (2017). DisGeNET: A Comprehensive Platform Integrating Information on Human Disease-Associated Genes and Variants. Nucleic Acids Res..

[36] Yang K., Wang R., Liu G., Shu Z., Wang N., Zhang R., Yu J., Chen J., Li X., Zhou X. (2019). HerGePred: Heterogeneous Network Embedding Representation for Disease Gene Prediction. IEEE J. Biomed. Health Inform..

[37] Wang X., Liu N., Han H., Shi C. (2021). Self-Supervised Heterogeneous Graph Neural Network with Co-Contrastive Learning. arXiv.

[38] Yang Z., Wu L., Wang A., Tang W., Zhao Y., Zhao H., Teschendorff A.E. (2017). DbDEMC 2.0: Updated Database of Differentially Expressed miRNAs in Human Cancers. Nucleic Acids Res..

